# Complete mitochondrial genome of *Scincella modesta* (Squamata: Scincidae)

**DOI:** 10.1080/23802359.2020.1713919

**Published:** 2020-01-16

**Authors:** Lian Chen, Youfu Lin, Ying Lin, Yaping Hu, Qi Xiao, Xu Zhou, Yan Liu, Hong Li

**Affiliations:** a College of Biology and the Environment, Nanjing Forestry University, Nanjing, China;; b Jiangsu Key Laboratory for Biodiversity and Biotechnology, College of Life Sciences, Nanjing Normal University, Nanjing, China;; c Ministry of Ecology and Environment of China, Nanjing Institute of Environmental Science, Nanjing, China;; d State Environmental Protection Scientific Observation and Research Station for Ecological Environment of Wuyi Mountains, Nanjing, China

**Keywords:** Mitochondrial genome, phylogenetic relationship, *Scincella modesta*

## Abstract

The first complete mitochondrial genome sequence was determined for an oviparous lizard, *Scincella modesta* (*Scincella,* Scincidae). The total length of the complete mitochondrial genome was 17,511 bp, encodes 13 protein-coding genes, 22 tRNAs, 2 rRNA genes, and 2 non-coding regions. The overall base composition of *S. modesta* is A: 31.9%, T: 27.2%, G: 14.5%, and C: 26.5%. Most of the *S. modesta* mitochondrial genes are encoded on the H-strand except for the ND6 gene and eight tRNA genes, which are encoded on the L-strand. Mrbayes and ML tree based on 13 protein-coding genes indicated that *S. vandenburghi* is the sister group of the *S. modesta* within the genus *Scincella.* The complete mitogenome sequence of *S. modesta* provided fundamental data for resolving phylogenetic and genetic problems related to genus *Scincella.*

The majority of lizard species in the family Scincidae are found in the subfamily Lygosominae. The lygosomine skinks of the genus *Scincella* contain small skinks comprising 35 species of small-sized ground lizards (Greer and Shea 2003; Linkem et al. 2011), but only 14 species can be found in China mainland. The slender forest skink (*Scincella modesta*) studied is a small-sized [to 55 mm snout-vent length (SVL)] oviparous diurnal sincid lizard that is widely distributed in the eastern and central provinces of China (Zhao and Adler 1993). Adult *S. modesta* are morphologically similar to a medium-sized sympatric viviparous skink (*Sphenomorphus indicus*) that they are often regarded wrongly as juveniles of the latter species. Despite the fact that it is taxonomically and zoogeographically well known, the biology and ecology of this species remain almost unknown. Most previous studies on *S. modesta* were about its sexual size dimorphism (Yang et al. 2012), reproductive traits and thermal biology (Li et al. 2012) and so on. In order to do so, further research on molecular levels about *S. modesta* and other skinks in the genus *Scincella*, it is necessary to analyze its complete mitochondrial genome.

The specimen of *S. modesta* was collected from Zijin mountain in Nanjing, Jiangsu province, China (N 32°3′48.35″, E 118°50′9.6″). The specimen is stored in the College of Life Sciences, Nanjing Normal University in China (Voucher No. 0216). Whole genomic DNA was extracted from the tail tissues of the specimens using the Animal Genomic DNA Extraction Kit (Roche, Germany). For amplification of the *S. modesta* mitochondrial genome, specific PCR primers were designed based on the mitochondrial genome sequences obtained from *Sphenomorphus incognitus* (MH329292). The complete mitochondrial (17,511 bp in length) genome of *S. modesta* (GenBank: MN786972) contained 22 tRNA genes, 2 rRNA genes (12S rRNA and 16S rRNA), 13 protein-coding genes, and 2 non-coding regions. In the 13 protein-coding genes: 12 protein-coding genes had ATG as the start codon, while *COXI* started with GTG. Five protein-coding genes (*ND1*, *ATP8*, *ATP6*, *ND4L, and ND5*) regarded TAA as stop codon; *ND2* and *CYTB* used TGG as stop codon; *ND6* used AGG as stop codon and *COXI* used AGA as stop codon; the others terminated with T as an incomplete stop codon, which was presumably completed as TAA by post-transcriptional polyadenylation (Anderson et al. 1981). The 22 tRNA genes range in size from 65 bp in tRNA^Cys^ to 75 bp in tRNALeu. The 12S rRNA and 16S rRNA genes were 976 bp and 1513 bp in size, respectively. Two ribosomal subunit genes were separated by the tRNA^Val^. Two non-coding regions with lengths of 2100 bp and 25 bp, respectively. The overall base composition of the H-strand for the mitogenome sequence of *S. modesta* was as follows: A = 31.9%, T = 27.2%, G = 14.5%, and C = 26.5%. Phylogenetic analysis revealed that there were evolutionary relationships within Scincidae (Figure 1). The three skink species showed a close phylogenetic relationship with other species in Scincidae, which clustered in a monophyletic group.

**Figure 1.**
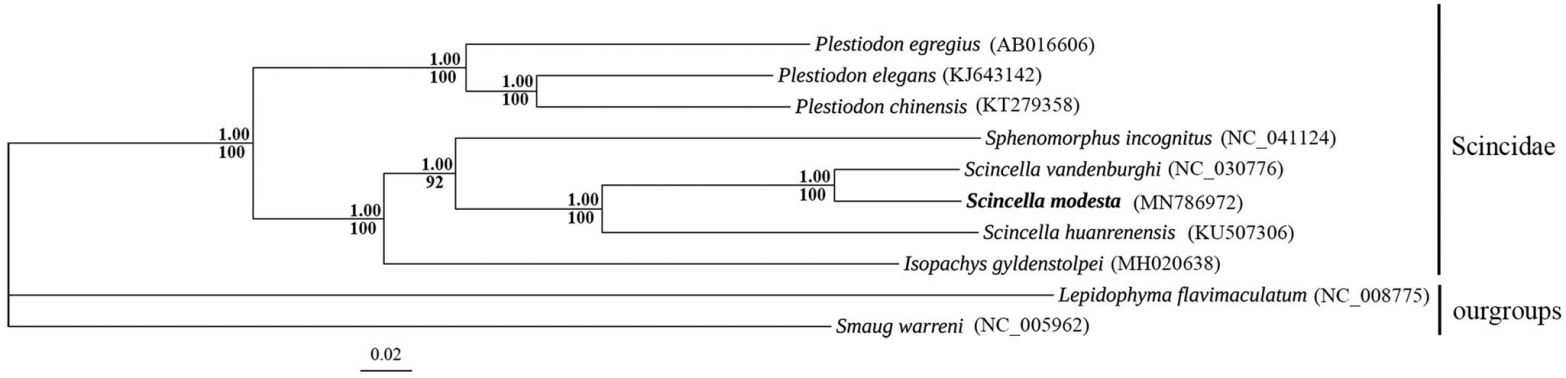
Phylogenetic tree inferred by maximum likelihood analyses and Bayesian inference based on PCG123 showing the relationship between *Scincella modesta* and other Scincidae mitochondrial genomes. GenBank accession numbers of mitogenome sequences used are shown in parentheses. Node labels indicate bootstrap values and posterior probabilities.

## References

[CIT0001] Anderson S, Bankier AT, Barrell BG, de Bruijn MH, Coulson AR, Drouin J, Eperon IC, Nierlich DP, Roe BA, Sanger F, et al. 1981. Sequence and organization of the human mitochondrial genome. Nature. 290:457. 7219534 10.1038/290457a0

[CIT0002] Greer AE, Shea G. 2003. Secondary temporal scale overlap pattern: a character of possible broad systematics importance in Sphenomorphine skinks. J Herpetol. 37(3):545–549.

[CIT0003] Linkem CW, Diesmos AC, Brown RM. 2011. Molecular systematics of the Philippine forest skinks (Squamata: Scincidae: Sphenomorphus): testing morphological hypotheses of interspecific relationships. Zool J Linnean Soc. 163(4):1217–1243. 10.1111/j.1096-3642.2011.00747.x PMC7165859 32336789

[CIT0004] Li H, Wang Z, Chen C, Ji X. 2012. Does the variance of incubation temperatures always constitute a significant selective force for origin of reptilian viviparity? Curr Zool. 58(6):812–819.

[CIT0005] Yang J, Sun YY, Fu TB, Xu DD, Ji X. 2012. Selection for increased maternal body volume does not differ between two Scincella lizards with different reproductive modes. Zoology. 115(4):199–206. 22749616 10.1016/j.zool.2012.01.004

[CIT0006] Zhao EM, Adler K. 1993. Herpetology of China. Oxford, Ohio, USA: Society for the Study of Amphibians and Reptiles.

